# Downstream Mammary and Extramammary Cascade Services and Spending Following Screening Breast Magnetic Resonance Imaging vs Mammography Among Commercially Insured Women

**DOI:** 10.1001/jamanetworkopen.2022.7234

**Published:** 2022-04-13

**Authors:** Ishani Ganguli, Nancy L. Keating, Nitya Thakore, Joyce Lii, Sughra Raza, Lydia E. Pace

**Affiliations:** 1 Harvard Medical School, Boston, Massachusetts; 2 Division of General Internal Medicine, Brigham and Women’s Hospital, Boston, Massachusetts; 3 Department of Health Care Policy, Harvard Medical School, Boston, Massachusetts; 4 Grossman School of Medicine, New York University, New York; 5 Department of Radiology, UMass Memorial Medical Center, Worcester, Massachusetts

## Abstract

**Importance:**

Increasing use of screening breast magnetic resonance imaging (MRI), including among women at low or average risk of breast cancer, raises concerns about resulting mammary and extramammary cascades (downstream services and new diagnoses) of uncertain value.

**Objective:**

To estimate rates of cascade events (ie, laboratory tests, imaging tests, procedures, visits, hospitalizations, and new diagnoses) and associated spending following screening breast MRI vs mammography among commercially insured US women.

**Design, Setting, and Participants:**

This cohort study used 2016 to 2018 data from the MarketScan research database (IBM Corporation), which includes claims and administrative data from large US employers and commercial payers. Participants included commercially insured women aged 40 to 64 years without prior breast cancer who received an index bilateral screening breast MRI or mammogram between January 1, 2017, and June 30, 2018. We used propensity scores based on sociodemographic, clinical, and utilization variables to match MRI recipients to mammogram recipients in each month of index service use. Data were analyzed from October 8, 2020, to October 28, 2021.

**Exposures:**

Breast MRI vs mammography.

**Main Outcomes and Measures:**

Mammary and extramammary cascade event rates and associated total and patient out-of-pocket spending in the 6 months following the index test.

**Results:**

In this study, 9208 women receiving breast MRI were matched with 9208 women receiving mammography (mean [SD] age, 51.4 [6.7] years). Compared with mammogram recipients, breast MRI recipients had 39.0 additional mammary cascade events per 100 women (95% CI, 33.7-44.2), including 5.0 additional imaging tests (95% CI, 3.8-6.2), 17.3 additional procedures (95% CI, 15.5-19.0), 13.0 additional visits (95% CI, 9.4-17.2), 0.34 additional hospitalizations (95% CI, 0.18-0.50), and 3.0 additional new diagnoses (95% CI, 2.5-3.6). For extramammary cascades, breast MRI recipients had 19.6 additional events per 100 women (95% CI, 8.6-30.7) including 15.8 additional visits (95% CI, 10.2-21.4) and no statistically significant differences in other events. Breast MRI recipients had higher total spending for mammary events ($564 more per woman; 95% CI, $532-$596), extramammary events ($42 more per woman; 95% CI, $16-$69), and overall ($1404 more per woman; 95% CI, $1172-$1636). They also had higher overall out-of-pocket spending ($31 more per woman; 95% CI, $6-$55).

**Conclusions and Relevance:**

In this cohort study of commercially insured women, breast MRI was associated with more mammary and extramammary cascade events and spending relative to mammography. These findings can inform cost-benefit assessments and coverage policies to ensure breast MRI is reserved for patients for whom benefits outweigh harms.

## Key Points

**Question:**

What is the prevalence of cascades of medical services and new diagnoses following screening breast magnetic resonance imaging (MRI) vs mammography among commercially insured US women?

**Findings:**

In this cohort study of 9208 women receiving MRI and 9208 propensity score–matched women receiving mammography, breast MRI recipients had higher rates of downstream mammary and extramammary visits, total and out-of-pocket spending, and for mammary findings specifically, higher rates of imaging tests, procedures, hospitalizations, and new diagnoses.

**Meaning:**

Screening breast MRI recipients experienced more mammary and extramammary cascade events and spending relative to mammogram recipients, with potential for harms to these patients and the health system.

## Teaser

This cohort study uses commercial claims data to estimate rates of cascade events and associated spending following screening breast magnetic resonance imaging (MRI) vs mammography among commercially insured women in the US.

## Introduction

Breast magnetic resonance imaging (MRI) is increasingly performed for breast cancer screening, typically in addition to mammographic screening.^1,2,3^ Breast MRI is more sensitive than mammography at identifying cancers,^4^ and although studies have not yet demonstrated that breast MRI screening reduces breast cancer mortality, in 2007, the American Cancer Society recommended screening MRI for all women with a lifetime risk of breast cancer exceeding 20%.^5^ Additionally, widespread breast density legislation in the US requires that patients be notified about their mammographic breast density and the potential role of supplemental imaging, such as MRI, despite uncertainty about whether supplemental MRI improves long-term outcomes for women with dense breasts. Such legislation, along with mandated insurance coverage for this supplemental imaging in some states,^6,7^ has further driven use of screening breast MRI.^8,9^ While the Society of Surgical Oncology, the American College of Physicians, and other groups recommend against breast MRI screening in women with low to average risk for breast cancer (eAppendix in the Supplement),^10^ such use may be common. A 2017 registry-based study in community settings found that 83% of screening breast MRI test were discordant with these guidelines.^11^

The increasing use of screening breast MRI, especially among women with low or average-risk for breast cancer, raises concerns about the extent to which these studies trigger downstream cascades of care that may have limited value and entail potential harm for patients.^12,13,14,15,16,17^ Breast MRI is less specific than mammography for breast pathology,^4,18^ resulting in more false-positive results and unnecessary biopsies.^19,20,21^ In addition, unlike mammography, breast MRI can reveal extramammary findings that may similarly prompt follow-up imaging and other services of unclear benefit. Single-site studies estimate that 10% to 34% of breast MRI tests show extramammary incidental findings.^17,22,23,24^ However, to our knowledge, no larger studies have examined the prevalence of or spending on downstream services that may follow. Further, extramammary cascades are rarely considered in breast MRI trials, cost-effectiveness analyses, guidelines, or shared decision-making discussions about breast cancer screening.^25,26^

Given growing use of screening breast MRI and the potential impact on patients and the health care system, it is important to characterize the national scope and associated spending on cascades following both mammary and extramammary findings. We used commercial claims data from across the US to compare women receiving breast MRI vs mammography screening on rates of downstream laboratory tests, imaging tests, procedures, visits, hospitalizations, and new diagnoses that could potentially follow mammary and extramammary findings on breast MRI, building on methods developed in prior work.^12,13,27^ We also compared total and patient out-of-pocket spending on cascade services and overall.

## Methods

This cohort study was considered exempt from review by the Harvard Medical School and Mass General Brigham institutional review boards. Informed consent was waived because patient data were deidentified. This study followed the Strengthening the Reporting of Observational Studies in Epidemiology (STROBE) reporting guideline. We used enrollment, outpatient, and inpatient data from January 1, 2016, to December 31, 2018, from the MarketScan research database (IBM Corporation), which includes medical claims from large employers and commercial payers in the US.

### Study Cohorts

#### Breast MRI and Mammography Cohorts

We identified all female members who had a bilateral breast MRI claim between January 1, 2017, and June 30, 2018; analysis was limited to the first MRI received during the study period. We further selected women who were aged 40 to 64 years on the date of the MRI, were continuously enrolled in their plan from 12 months before the MRI through 6 months after the MRI, and did not have another breast MRI claim or diagnostic mammography claim in the preceding 12 months. We further excluded women with any claim including a breast cancer diagnosis code in the preceding 12 months of the index MRI or on the date of the index MRI (eTable 1 in the Supplement), and women with any claim including a diagnostic code for managing breast implants, any cancer surveillance, or diagnostic imaging on the date of the index MRI. We took these steps based on prior literature to optimize the likelihood of identifying a screening MRI since bilateral breast MRI procedure codes do not specify screening vs diagnostic indication.^28,29^ Among women who did not have a breast MRI, we identified those who had a bilateral screening mammogram claim between January 1, 2017, and June 30, 2018; were aged 40 to 64 years on the date of the mammogram; were continuously enrolled from 12 months before the mammogram through 6 months after the mammogram; did not have a breast MRI claim in the preceding 12 months; and did not have a claim with a breast cancer diagnosis code in the preceding 12 months or on the date of the index mammogram.

#### Propensity Score Matching

We used propensity scores to match women who received breast MRI with similar women who received mammography based on variables observable in the data that might influence the decision to obtain MRI, informed by published literature, clinical guidelines, and experience.^11,19,28,30^ Specifically, we used 1:1 greedy matching without replacement to match 1 mammogram recipient to each MRI recipient with the same month of index service use based on the propensity score for that month using models that included: (1) age (continuous); (2) US census division (out of 9 total)^31^; (3) combined comorbidity score in the year before the index test^32^; (4) prior utilization (total outpatient health care spending in the prior 12 months); (5) health plan type (Table 1), a binary indicator for whether the index test occurred on or after breast density legislation was enacted in the member’s state of residence^33^; and (6) any diagnosis or procedure code in the prior 12 months suggesting higher than average risk of breast cancer or of breast MRI receipt (ie, family history of breast cancer, *BRCA1* or *BRCA2* mutation, dense breasts, history of chest irradiation, high-risk nonmalignant breast lesion, benign breast disease, or breast biopsy) (eTable 2 in the Supplement). We used standardized differences to assess the balance between the MRI and mammography cohorts before and after propensity matching.^34^

**Table 1.** Characteristics of the Study Population Before and After Propensity Matching

Characteristic	Before matching			After matching		
	Patients, No. (%)		Standardized difference^a^	Patients, No. (%)		Standardized difference^a^
	Breast MRI	Mammography		Breast MRI	Mammography	
No.	9351	1 841 424		9208	9208	
Age, mean (SD)	51.5 (6.6)	52.7 (6.8)	−0.190	51.5 (6.6)	51.3 (6.9)	0.020
Census division						
New England	583 (6.2)	72 889 (4.0)	0.103	575 (6.2)	587 (6.4)	−0.005
Middle Atlantic	1529 (16.4)	208 252 (11.3)	0.146	1487 (16.2)	1557 (16.9)	−0.020
East North Central	1266 (13.5)	269 560 (14.6)	−0.032	1238 (13.4)	1231 (13.4)	0.002
West North Central	378 (4.0)	89 830 (4.9)	−0.041	373 (4.1)	383 (4.2)	−0.006
South Atlantic	1786 (19.1)	399 932 (21.7)	−0.065	1766 (19.2)	1690 (18.4)	0.021
East South Central	378 (4.0)	116 691 (6.3)	−0.104	374 (4.1)	392 (4.3)	−0.010
West South Central	478 (5.1)	160 245 (8.7)	−0.142	474 (5.2)	470 (5.1)	0.002
Mountain	426 (4.6)	70 284 (3.8)	0.037	423 (4.6)	443 (4.8)	−0.010
Pacific	1164 (12.5)	165 723 (9.0)	0.112	1149 (12.5)	1178 (12.8)	−0.009
Unknown census division	1363 (14.6)	288 018 (15.6)	−0.030	1349 (14.7)	1277 (13.9)	0.022
Breast density law present in state	7633 (81.6)	1 448 138 (78.6)	0.075	7514 (81.6)	7546 (82.0)	−0.009
Breast diagnoses						
Benign breast disease	3070 (32.8)	64 523 (3.5)	0.823	2945 (32.0)	3042 (33.0)	−0.023
Histologic high-risk benign breast lesion	695 (7.4)	3773 (0.20)	0.384	632 (6.9)	514 (5.6)	0.053
History of chest irradiation	49 (0.52)	1697 (0.09)	0.078	48 (0.52)	68 (0.74)	−0.028
Dense breasts	1796 (19.2)	25 436 (1.4)	0.614	1699 (18.5)	1514 (16.4)	0.053
Family history of breast cancer or genetic susceptibility	4747 (50.8)	26 414 (1.4)	1.358	4611 (50.1)	4661 (50.6)	−0.011
History of breast biopsy	527 (5.6)	7045 (0.38)	0.312	473 (5.1)	443 (4.8)	0.015
Prior outpatient spending, mean (SD)	6808.57 (12 626.30)	4559.35 (12 298.96)	0.180	6742.28 (12 639.67)	7776.66 (18 710.75)	−0.060
Plan type						
Consumer-driven health plan	984 (10.5)	259 621 (14.1)	−0.109	974 (10.6)	971 (10.6)	0.001
Comprehensive plan	274 (2.9)	59 071 (3.2)	−0.016	269 (2.9)	291 (3.2)	−0.014
Exclusive provider organization plan	71 (0.76)	15 023 (0.82)	−0.007	68 (0.74)	89 (0.97)	−0.025
High-deductible health plan	787 (8.4)	163 477 (8.9)	−0.016	781 (8.5)	759 (8.2)	0.009
Health maintenance organization plan	1022 (10.9)	213 729 (11.6)	−0.022	1010 (11.0)	1064 (11.6)	−0.019
Point-of-Service Plan	606 (6.5)	115 378 (6.3)	0.009	597 (6.5)	614 (6.7)	−0.008
Capitated point-of-service plan	170 (1.8)	23 188 (1.3)	0.45	168 (1.8)	161 (1.8)	0.005
Preferred provider organization plan	5239 (56.0)	957 983 (52.0)	0.081	5153 (56.0)	5044 (54.8)	0.024
Unknown	198 (2.1)	33 954 (1.8)	0.020	188 (2.0)	215 (2.3)	−0.020
Combined comorbidity score, mean (SD)	0.41 (1.1)	0.26 (1.00)	0.150	0.41 (1.05)	0.45 (1.2)	−0.030

^a^ Standardized differences measure the balance in baseline covariates between 2 groups. Values less than 0.10 demonstrate that the groups are well-balanced.

#### Index and Cascade Events

We defined the index event as the index breast MRI (for the breast MRI cohort) or the index mammogram (for the mammography cohort). We defined the cascade period as the 6 months starting the day after the index event. We used literature^15,16,17,23^ and clinical expert review (S.R., I.G., N.K., L.P.) to identify potential cascade events: all laboratory tests, imaging tests, procedures, visits, hospitalizations, and new diagnoses that may plausibly follow from a mammary or extramammary finding on breast MRI (eTables 3-7 in the Supplement). We categorized laboratory tests, imaging tests, procedures, visits, and diagnoses as related to mammary or nonmammary findings; since billing codes for the laboratory tests likely to follow mammary findings (ie, pathology and cytology) do not have sufficient specificity to the breast, we grouped these events as a separate category (pathology and cytology). For laboratory tests (including pathology and cytology), imaging tests, and procedures, we counted only 1 event within a given subcategory on a given day of service (eg, if there were 2 billing codes for breast ultrasonography on the same day, we counted 1 ultrasonography test, but if there were billing codes for breast ultrasonography and breast MRI on the same day, we counted 2 unique events). Hospitalizations were identified based on having a cascade-relevant principal diagnosis (based on new diagnoses described above). The authors grouped diagnosis codes into subcategories (eg, renal cyst), and each given subcategory could only be counted once. A diagnosis was defined as new if it was included on any inpatient claim during hospitalization or on 2 outpatient visit claims during the cascade period but not during the 12 months before the index event.

#### Spending

Using inpatient and outpatient files, we measured total spending (ie, all allowable payments) and out-of-pocket spending (ie, the sum of copayments, coinsurance, and deductibles) for mammary and extramammary cascade-attributable services during the 6-month cascade period. We then assessed total spending and out-of-pocket spending for all services received during the cascade period. We estimated mean total and out-of-pocket spending on the index breast MRI and index mammograms among the respective recipients. To account for the non-independence of index and cascade service out-of-pocket spending (eg, index event out-of-pocket spending could affect cascade out-of-pocket spending by contributing to deductible limits), we also estimated out-of-pocket spending for mammary cascades, extramammary cascades, and all services while including the cost of the index MRI or mammogram.

### Statistical Analysis

We estimated cascade event rates and spending for the breast MRI and mammography groups, Winsorizing estimates at 1%. We then created Poisson regression models to estimate cascade event rates and log-linear regression models to estimate spending during the cascade period for each cohort. The Poisson models were adjusted for potential overdispersion by scaling according to the Pearson residuals. We then estimated cascade event rate and spending differences between the MRI and mammography groups, using *t* values to determine CIs. Since mammogram findings are limited to the breast, extramammary events following index mammograms were captured to represent baseline service rates and permit quantification of extramammary cascades in the MRI group. In our primary analysis, we did not count mammograms that occurred during the cascade period as mammary cascade events since they may instead represent standalone subsequent screening events; we performed a sensitivity analysis in which we repeated our estimates of mammary cascades including mammograms as cascade events.

To test the assumption that additional cascade events in the breast MRI group are plausibly causally linked to the index events rather than due to selection effects (eg, if women who receive breast MRI also use more health care overall), we performed a falsification test in which we repeated the cascade analyses with a service, knee x-ray, that would not plausibly follow from a breast MRI. Finally, we estimated the proportion of breast MRI recipients who had any potential mammary cascade event, any potential extramammary cascade event, both, or neither. We described these as potential cascade events because they included some commonly performed medical services, and we could not confirm in this observational, claims-based analysis whether the event followed causally from the index screening test.

We performed analyses using SAS software, version 9.4 (SAS Institute, Inc). Reported *P* values were 2 sided and *P* < .05 represented statistical significance. Data were analyzed from October 8, 2020, to October 28, 2021.

## Results

Before matching, women receiving breast MRI were more likely to be younger, to have prior breast-related diagnoses (such as benign breast disease) and prior breast biopsy, and to reside in New England or Middle Atlantic regions than women receiving mammography (Table 1). After propensity score matching, there were 18 416 women in our cohort (mean [SD] age, 51.4 [6.7] years). Characteristics of the breast MRI (n = 9208) and mammography (n = 9208) groups were well-balanced, with standardized differences for all covariates less than 0.1.

### Cascade Event Rates

When examining mammary cascade events, breast MRI recipients had 39.0 additional cascade events per 100 women (95% CI, 33.7 to 44.2) (Table 2). These included additional imaging tests (5.0 per 100 women; 95% CI, 3.8 to 6.2), procedures (17.3; 95% CI, 15.5 to 19.0), specialist visits (13.0; 95% CI, 9.4 to 17.2), hospitalizations (0.34; 95% CI, 0.18 to 0.50), and new diagnoses (3.0; 95% CI, 2.5 to 3.6) compared with mammogram recipients (Table 2). Breast MRI recipients also had higher rates of pathology and cytology tests (14.1; 95% CI, 12.3 to 15.9). In the sensitivity analysis including mammograms as cascade events, the rate of additional imaging tests for MRI recipients was higher (eTable 8 in the Supplement). When examining extramammary cascade events, breast MRI recipients had 19.6 additional services per 100 women (95% CI, 8.6 to 30.7), including 15.8 additional visits (95% CI, 10.2 to 21.4). There were no statistically significant differences between the breast MRI and mammography groups in rates of laboratory tests, imaging tests, procedures, hospitalizations, and new diagnoses. The falsification test showed that there were 4.2 knee x-rays per 100 in the MRI cohort vs 4.3 knee x-rays per 100 in the mammogram cohort, with a nonsignificant difference of −0.13; (95% CI, −0.81 to 0.55).

**Table 2.** Cascade Event Rates Among Screening Breast MRI vs Mammography Recipients in the 6 Months Following the Screening Test

Characteristics	Event rate per 100 members		Breast MRI cascade-attributable event rate per 100 members (95% CI)
	Breast MRI (n = 9208)	Mammography (n = 9208)	
Mammary			
All mammary cascade events	134.1	95.1	39.0 (33.7 to 44.2)
Imaging tests	18.1	13.1	5.0 (3.8 to 6.2)
Procedures	22.4	5.1	17.3 (15.5 to 19.0)
Visits	89.3	76.0	13.3 (9.4 to 17.2)
Hospitalizations	0.38	0.04	0.34 (0.18 to 0.50)
New diagnoses	3.9	0.9	3.0 (2.5 to 3.6)
Extramammary			
All extramammary cascade events	304.5	284.8	19.6 (8.6 to 30.7)
Laboratory tests	114.9	111.8	3.2 (−2.2 to 8.5)
Imaging tests	33.3	33.7	−0.45 (−2.7 to 1.8)
Procedures	10.3	9.4	0.97 (−0.08 to 2.0)
Visits	141.0	125.2	15.8 (10.2 to 21.4)
Hospitalizations	0.27	0.38	−0.11 (−0.28 to 0.06)
New diagnoses	4.7	4.4	0.29 (−0.53 to 1.11)
Other^a^			
Pathology/cytology	35.8	21.7	14.1 (12.3 to 15.9)

Abbreviation: MRI, magnetic resonance imaging.

^a^ Since billing codes for the laboratory tests likely to follow mammary findings (ie, pathology and cytology) do not have sufficient specificity to identify breast vs other tissue sites for biopsy or cytology samples, we grouped these events as a separate category and did not include them in counts of aggregate cascade event or spending estimates.

### Spending

Breast MRI recipients had higher total spending on mammary cascades ($564; 95% CI, $532-$596) and on extramammary cascades ($42; 95% CI, $16-$69) (Figure). Breast MRI recipients had higher out-of-pocket spending on mammary cascades and slightly lower out-of-pocket spending on extramammary cascades. However, when we included the cost of the index event in the cascade estimates (because index event out-of-pocket spending for MRI and mammography could affect cascade out-of-pocket spending by contributing to deductible limits), we found that breast MRI recipients had higher out-of-pocket spending for both mammary services ($305; 95% CI, $297-$313) and extramammary services ($254; 95% CI, $244-$263) (eTable 9 in the Supplement). When considering spending on all services received in the cascade period, breast MRI recipients had higher mean total overall spending ($1404 more per woman; 95% CI, $1172-$1636) and higher mean total out-of-pocket spending ($31 more per woman; 95% CI, $6-$55) (Figure).

**Figure.**
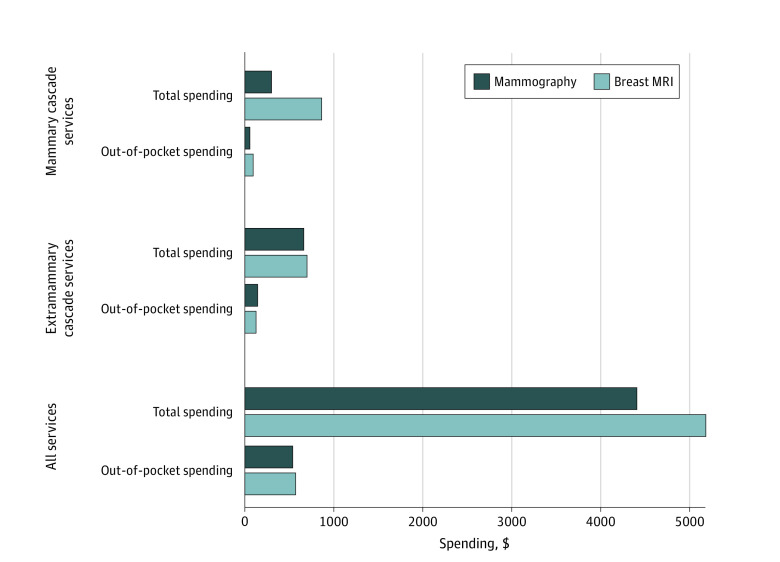
Total and Out-of-Pocket Spending on Mammary Cascade Services, Extramammary Cascade Services, and All Services Among Screening Breast MRI vs Mammography Recipients in the 6 Months Following the Screening Test

Among the 7015 (76.2%) breast MRI recipients with any potential cascade event (which included services that are commonly performed for a variety of indications), 338 (3.7%) had a potential mammary cascade event, 1949 (21.2%) had a potential extramammary cascade event, and 4728 (51.4%) had both.

## Discussion

In this national study of commercially insured women aged 40 to 64 years, we found that relative to women undergoing breast cancer screening with mammography only, women undergoing screening breast MRI experienced additional cascade services related to both mammary and extramammary findings. Breast MRI recipients had additional mammary imaging tests, procedures, visits, hospitalizations, and new diagnoses, additional extramammary visits, and additional pathology and cytology tests, with relatively modest associated additional overall and out-of-pocket spending.

We found that breast MRI was associated with substantially higher rates of cascades from mammary findings than mammograms, which would be expected given MRI’s higher sensitivity and lower specificity for breast cancer detection relative to mammography and well-documented higher rates of false-positive results and biopsies, especially among women without known breast cancer.^19,21^ Our results further demonstrate higher rates of office visits and hospitalizations for mammary diagnoses following MRI, which to our knowledge have not been previously shown.

Our estimates of nonmammary cascades following breast MRI in a large, population of women who were commercially insured build on single institution studies on this topic.^17,22,23,24^ European studies have documented incidental findings in 10% to 34% of breast MRI tests.^23,24^ In a US study of 327 breast cancer patients, Padia et al^17^ found that 11% experienced 1 or more incidental extramammary findings, with many of those women receiving additional imaging (51%), laboratory testing (9%), physician referrals (6%), and biopsies (6%). None of those incidental findings turned out to be malignancies. Niell et al^22^ noted extramammary findings in 17% of 2324 examined breast MR images, of which 4% were recommended for follow up imaging—most often for hepatic lesions—and only 0.4% led to a clinically important finding. Our study showed that women receiving MRI had higher rates of total extramammary events and specialist visits than women receiving mammograms, while small differences in other extramammary events did not reach statistical significance.

We also found that MRI receipt was associated with more cascade-attributable and overall total and out-of-pocket spending than mammography receipt. When considering extramammary cascade spending (for which mammogram recipients represented baseline spending rates on these services), compared with mammogram recipients, MRI recipients had $42 additional total spending and $9 less out-of-pocket spending.^22^ This negative result is likely explained by MRI recipients being more likely to reach their deductible because of the MRI itself. In support of this possibility, when we included index event cost in the comparison of extramammary cascade spending, MRI recipients spent an additional $254 out of pocket. These results use actual payer reimbursements and build on prior single-site studies that estimated spending based on standardized Medicare reimbursement rates. Niell et al^22^ estimated a mean of $16 in spending across all MRI recipients for within-site follow-up imaging tests alone for extramammary findings. Padia et al^17^ estimated a mean of $328 spent on downstream extramammary laboratory tests, imaging, and biopsies across all women who had incidental findings.

While our estimates of total additional spending are modest, they add up across the population. A claims-based study^28^ showed about 0.18% of all US women aged 40 to 64 received screening MRI in 2016 (0.19% among women aged 40-49; 0.17% among women aged 50-64), with current rates likely to be higher. Extrapolating to 52.6 million women aged 40 to 64 based on 2020 US census data, this would entail about $53.4 million in additional total spending on mammary cascades and $4.0 million on extramammary cascades nationwide. Cost-effectiveness analyses of breast MRI and mammography to date have not included the downstream costs of MRI extramammary findings in their assessments and are overall less comprehensive in their examination of events.^25,35,36,37^ Differences in out-of-pocket spending from cascades are critical to share with women when making decisions about breast cancer screening approaches, especially given growing enrollment in high deductible health plans.^29,38^

Our findings about additional mammary and extramammary cascade events associated with MRI contribute to understanding the potential benefits and harms of these studies. MRI screening has been associated with earlier-stage breast cancer diagnoses and may have value in women at elevated breast cancer risk. However, MRI also prompts overdiagnosis and overtreatment of breast cancer.^39^ Repeated exposure to gadolinium contrast is another concern that is not well understood.^40^ Our findings regarding downstream cascades and spending associated with MRI further underscore the importance of identifying patients who are most likely to derive benefit from these studies.

### Limitations

This study has limitations inherent to claims-based analyses. Although use of administrative data allowed us to examine a large population across the US, these data lacked clinical detail to confirm intentions behind billed services or to determine if downstream events followed causally from the index event. Specifically, bilateral breast MRI codes do not distinguish between screening and diagnostic indications, although we took several analytic steps to exclude diagnostic MRI consistent with prior literature.^28,29^ In addition, potential cascade events necessarily include services commonly performed for various indications, so while we report these rates, our main conclusions focus on additional event rates in the breast MRI group compared with the mammography group. The falsification test offers further reassurance that the additional cascade events reported may plausibly be associated with the index event. We also could not account for all potential confounders associated with breast MRI receipt, such as detailed risk scores, race, facility or clinician characteristics, or socioeconomic status.^41^ For example, clinicians may be more likely to use billing codes for breast cancer risk factors when ordering MRI to facilitate insurance coverage, and thus these risk factors may be less often identified for the mammography cohort. We expect such differences would bias our results to the null.

Additionally, we did not observe the clinical impact of the cascade events and do not place judgments on the value of individual cascade events. While prior work suggests that some of these downstream services had minimal benefit for patients,^17,22^ some incidental extramammary findings from breast MRI—such as a newly diagnosed lung cancer—could have improved health. Regarding mammary services following breast MRI, although some women will benefit from earlier detection of breast cancer, the proportion experiencing low-value mammary services from false-positive results is likely to be high in the substantial number of women receiving MRI who do not have elevated breast cancer risk.^11^ Finally, we studied women aged 40 to 64 years with commercial insurance and further study is needed to determine whether our results are generalizable to older women or women with other types of health insurance.

## Conclusions

Our study provides a detailed national picture of downstream services and new diagnoses following breast MRI relative to mammography, including extramammary events and spending that have not been well-characterized previously. The additional testing and related insurer and out-of-pocket spending has important implications for understanding the benefits and harms of MRI screening at the population level and for assisting women in shared decision making about MRI screening. Our findings underscore the importance of avoiding screening breast MRI in low or average-risk women for whom potential harms from screening outweigh potential benefits.

## Notes

Supplement. **eAppendix.** Relevant Choosing Wisely Recommendation **eTable 1. **Diagnosis and Procedure Codes Used in MRI and Mammography Cohort Selection **eTable 2. **Diagnosis and Procedure Codes for Propensity to Receive Breast MRI **eTable 3. **Procedure Codes for Cascade Laboratory Tests **eTable 4.** Procedure Codes for Cascade Imaging Tests **eTable 5.** Procedure Codes for Cascade Procedures **eTable 6. **Procedure Codes for Cascade Visits and Specialty Codes for Cascade Specialty Visits **eTable 7. **Diagnosis Codes for Cascade New Diagnoses **eTable 8.** Cascade Event Rates and Total and Out-of-Pocket Spending on Mammary Cascade Services Among Screening Breast MRI vs Mammography Recipients in the 6 Months Following the Screening Test, Sensitivity Analysis Including Mammograms as Cascade Events **eTable 9. **Total and Out-of-Pocket Spending on Mammary Cascade Services, Extra-Mammary Cascade Services, and All Services Among Screening Breast MRI vs Mammography Recipients in the 6 Months Following the Screening Test, Including Spending on the Index Event
