# Supplementary material for: Downstream Mammary and Extramammary Cascade Services and Spending Following Screening Breast Magnetic Resonance Imaging vs Mammography Among Commercially Insured Women
Source: JAMA Netw Open. 2022 Apr 13;5(4):e227234. doi: 10.1001/jamanetworkopen.2022.7234 (PMC9008498; doi:10.1001/jamanetworkopen.2022.7234)
Supplement: Supplement. — eAppendix. Relevant Choosing Wisely Recommendation eTable 1. Diagnosis and Procedure Codes Used in MRI and Mammography Cohort Selection eTable 2. Diagnosis and Procedure Codes for Propensity to Receive Breast MRI eTable 3. Procedure Codes for Cascade Laboratory Tests eTable 4. Procedure Codes for Cascade Imaging Tests eTable 5. Procedure Codes for Cascade Procedures eTable 6. Procedure Codes for Cascade Visits and Specialty Codes for Cascade Specialty Visits eTable 7. Diagnosis Codes for Cascade New Diagnoses eTable 8. Cascade Event Rates and Total and Out-of-Pocket Spending on Mammary Cascade Services Among Screening Breast MRI vs Mammography Recipients in the 6 Months Following the Screening Test, Sensitivity Analysis Including Mammograms as Cascade Events eTable 9. Total and Out-of-Pocket Spending on Mammary Cascade Services, Extra-Mammary Cascade Services, and All Services Among Screening Breast MRI vs Mammography Recipients in the 6 Months Following the Screening Test, Including Spending on the Index Event [file jamanetwopen-e227234-s001.pdf]

Supplemental Online Content

Ganguli I, Keating NL, Thakore N, Lii J, Raza S, Pace LE. Downstream mammary and extramammary cascade services and spending following screening breast magnetic resonance imaging vs mammography among commercially insured women. *JAMA Netw Open*. 2022;5(4):e227234. doi:10.1001/jamanetworkopen.2022.7234

eAppendix. Relevant Choosing Wisely Recommendation

eTable 1. Diagnosis and Procedure Codes Used in MRI and Mammography Cohort Selection

eTable 2. Diagnosis and Procedure Codes for Propensity to Receive Breast MRI

eTable 3. Procedure Codes for Cascade Laboratory Tests

eTable 4. Procedure Codes for Cascade Imaging Tests

eTable 5. Procedure Codes for Cascade Procedures

eTable 6. Procedure Codes for Cascade Visits and Specialty Codes for Cascade Specialty Visits

eTable 7. Diagnosis Codes for Cascade New Diagnoses

eTable 8. Cascade Event Rates and Total and Out-of-Pocket Spending on Mammary Cascade Services among Screening Breast MRI vs Mammography Recipients in the 6 Months Following the Screening Test, Sensitivity Analysis including Mammograms as Cascade Events

eTable 9. Total and Out-of-Pocket Spending on Mammary Cascade Services, Extra-Mammary Cascade Services, and All Services Among Screening Breast MRI vs Mammography Recipients in the 6 Months Following the Screening Test, Including Spending on the Index Event

This supplemental material has been provided by the authors to give readers additional information about their work.

© 2022 Ganguli I et al. *JAMA Network Open.*

Appendix 1. Relevant Choosing Wisely Recommendation

Society of Surgical Oncology: **Don't routinely use breast MRI for breast cancer screening in average risk women.**
MRI screening should be reserved for those at increased risk. Women considered at high risk include: known BRCA gene mutation carriers; untested first-degree relatives of known BRCA gene mutation carriers; those with a lifetime risk exceeding 20% as measured by risk-assessment tools based primarily on family history of breast cancer; and those with a clinical history associated with a significant risk for breast cancer, including women who received mantle radiation before the age of 30. MRI for screening after treatment for breast cancer is not indicated in women who would otherwise be considered average risk.
Source: www.choosingwisely.org/clinician-lists/sso-breast-mri-to-screen-average-risk-women/

© 2022 Ganguli I et al. *JAMA Network Open.*

eTable 1. Diagnosis and Procedure Codes Used in MRI and Mammography Cohort Selection

Billing Code Type	Code
Bilateral breast MRI	77059, C8906, C8907, C8908<br>**Exclude if:**<br>Diagnostic mammography (77066, G0204, G0279) or bilateral breast MRI in preceding 12 months<br>Breast cancer (C50.XXX, D05.1X, Z85.3, Z86.000) in preceding 12 months or on index MRI<br>Secondary breast cancer (198.81, C79.81) on index MRI<br>Any neoplasm (140.XX-239.XX; C00.X-D49.X) on index MRI<br>Device complications (996.54, 996.69, 996.79, T854, T857, T858, T868) on index MRI<br>Breast implant (793.8X, R92.X (Except, 793.82, R92.2 for dense breast)) on index MRI
Bilateral screening mammogram*	77067, G0202, 77063<br>**Exclude if:**<br>Bilateral breast MRI in preceding 12 months<br>Breast cancer (C50.XXX, D05.1X, Z85.3, Z86.000) in preceding 12 months or on index MRI

*includes tomosynthesis; only counted as a single service if more than one of these coded on the same day

© 2022 Ganguli I et al. *JAMA Network Open.*

eTable 2. Diagnosis and Procedure Codes for Propensity to Receive Breast MRI

Category	Codes (CPT and ICD-10)
Family history of breast cancer	Z80.3
BRCA1/2 mutation	Z15.01
Breast density	R92.2
History of chest irradiation (including history of Hodgkin's Lymphoma)	Z92.3, Z85.71
High-risk non-malignant breast lesion (atypical ductal hyperplasia, atypical lobular hyperplasia, lobular carcinoma in situ, flat epithelial atypia)	N60.8X, N60.9X, D05.0X, D48.6X
Benign breast disease (breast cyst, diffuse cystic mastopathy, fibroadenosis of breast, fibrosclerosis of breast, Mammary Duct ectasia, hypertrophy of breast, mammographic microcalcification, abnormal and inconclusive findings on diagnostic imaging)	D24.X, N60.0X, N60.1X, N60.2X, N60.3X, N60.4X, N62, D05.8X, D05.9X, R92.0, R92.1, R92.8
Breast biopsy	19000, 19001, 19100, 19102, 19081-19086, 19120, 19125, 19126, 19101, 0HBT0ZZ, 0HBT3ZZ 0HBT0ZX, 0HBT3ZX, 0HBU0ZZ, 0HBU3ZZ, 0HBU0ZX, 0HBU3ZX 0HBV0ZZ, 0HBV3ZZ, 0HBV0ZX, 0HBV3ZX, 0HBW0ZZ, 0HBW3ZZ 0HBWXZZ, 0HBW0ZX, 0HBW3ZX, 0HBWXZX 0HBX0ZZ, 0HBX3ZZ, 0HBXXZZ 0HBX0ZX, 0HBX3ZX, 0HBXXZX

© 2022 Ganguli I et al. *JAMA Network Open.*

eTable 3. Procedure Codes for Cascade Laboratory Tests

	**Event type**	**Codes (CPT)**
**Extramammary**	Complete blood count	85025
	Complete blood count with differential	85027
	Basic metabolic panel	80048
	Complete metabolic panel	80053
	Hepatic panel	80076
	Thyroid function panel	84443
**Other**	Cytopathology	88172-3, 88177, 88305, 88307, 88309, 88104, 88106, 88108, 88112, 88160-2

© 2022 Ganguli I et al. *JAMA Network Open.*

eTable 4. Procedure Codes for Cascade Imaging Tests

	Event type	Codes (CPT and ICD-10)
**Mammary**	Breast ultrasound	76641-2, BH41ZZZ, BH40ZZZ, BH42ZZZ
	Mammogram (only considered as cascade event in sensitivity analysis)	*77051-2, 77055-7, 77065-7, 76376-7, 77061-3,<br>G0202, G0204, G0206, G0279,<br>BH00ZZZ, BH01ZZZ, BH02ZZZ*
	Breast MRI	77058-9, 76498, 77046-9, C8903, C8904, C8905, C8906, C8907, C8908, S8042,<br>BH30Y0Z, BH30YZZ, BH30ZZZ, BH31Y0Z, BH31YZZ, BH31ZZZ, BH32Y0Z, BH32YZZ, BH32ZZZ
	Nuclear breast imaging	78800-04, A4641, A4642, A9500, CH1YYZZ, CH2YYZZ, CH101ZZ, CH10SZZ, CH10YZZ, CH111ZZ, CH11SZZ, CH11YZZ, CH121ZZ, CH12SZZ, CH12YZZ, CH201ZZ, CH20SZZ, CH20YZZ, CH211ZZ, CH21SZZ, CH21YZZ, CH221ZZ, CH22SZZ, CH22YZZ
	Thermography	93740, 4A0ZXKZ
**Extra-mammary**	Thoracic cavity CT	71260, 71250, 71270, BW2400Z, BW240ZZ, BW2410Z, BW241ZZ, BW24Y0Z, BW24YZZ, BW24ZZZ, BW2500Z, BW250ZZ, BW2510Z, BW251ZZ, BW25Y0Z, BW25YZZ, BW25ZZZ
	Abdominal CT	74150, 74160, 74170, BW2000Z, BW200ZZ, BW2010Z, BW201ZZ, BW20Y0Z, BW20YZZ, BW20ZZZ
	Abdomen/pelvis CT	74176, 74177, 74178, BW2100Z, BW210ZZ, BW2110Z, BW211ZZ, BW21Y0Z, BW21YZZ, BW21ZZZ
	Pelvic CT	72192, 72193, 72194, BW2G00Z, BW2G0ZZ, BW2G10Z, BW2G1ZZ, BW2GY0Z, BW2GYZZ, BW2GZZZ, BR2C0ZZ, BR2C1ZZ, BR2CYZZ, BR2CZZZ
	Abdominal MRI	74181, 74182, 74183, BW30Y0Z, BW30YZZ, BW30ZZZ
	Pelvic MRI	72195, 72196, 72197, BR3CY0Z, BR3CYZZ, BR3CZZZ
	Chest ultrasound	76604, BH4BZZZ
	Retroperitoneal /pelvic/abdomen and pelvic ultrasound	76770, 76775, 76856, 76857, BW4GZZZ, BW41ZZZ
	Rib x-ray	71110-1, 71101, 71100, 71120, 71130, BP0XZZZ, BP0YZZZ
	Clavicle x-ray	73000, BP04ZZZ, BP05ZZZ
	Shoulder x-ray	73020, 73030, 73040, BP080ZZ, BP081ZZ, BP08YZZ, BP08ZZZ, BP090ZZ, BP091ZZ, BP09YZZ, BP09ZZZ
	Scapula x-ray	73010, BP06ZZZ, BP07ZZZ
	Sternum x-ray	BR0HZZZ
	Sacrum and coccyx x-ray	BR0FZZZ
	Spine x-ray	72020, BR0GZZ1, BR0GZZZ
	Spine, cervical x-ray	72040, 72050, 72052, BR00ZZ1, BR00ZZZ, BR010ZZ, BR011ZZ, BR01YZZ, BR01ZZZ
	Spine, thoracic x-ray	72070, 72072, 72074, BR07ZZ1, BR07ZZZ, BR020ZZ, BR021ZZ, BR02YZZ, BR02ZZZ
	Spine, thoracic and lumbar x-ray	72080, 72081, 72082, 72083, 72084,

© 2022 Ganguli I et al. *JAMA Network Open.*

	Event type	Codes (CPT and ICD-10)
	Spine, lumbosacral x-ray	72100, 72110, 72114, 72120, BR09ZZ1, BR09ZZZ, BR030ZZ, BR031ZZ, BR03YZZ, BR03ZZZ
	Pelvis x-ray	72170, 72190, BR0CZZZ
	All long bones x-ray	BW0BZZZ
	Whole skeleton x-ray	BW0LZZZ
	Nuclear medicine scans including bone, neck, chest, abdomen	78300, 78305, 78306, 78315, 78320, CW131ZZ, CW231ZZ, CW141ZZ, CW241ZZ, CW161ZZ, CW261ZZ, CW101ZZ CW201ZZ
	Chest x-ray	71045, 71046, 71047, 71048, 71020, 71030, 71034, 71035, 71010, BW03ZZZ
	Cardiac MRI	75557, 75561, 75565,
	Cardiac CT	75571-4
	Renal angiography	36251-4, BT110ZZ, BT111ZZ, BT11YZZ, BT11ZZZ, BT120ZZ, BT121ZZ, BT12YZZ, BT12ZZZ, BT130ZZ, BT131ZZ, BT13YZZ, BT13ZZZ, B416010, B4160ZZ, B416110, B4161ZZ, B416Y10, B416YZZ, B416ZZZ, B417010, B4170ZZ, B417110, B4171ZZ, B417Y10, B417YZZ, B417ZZZ, B418010, B4180ZZ, B418110, B4181ZZ, B418Y10, B418YZZ, B418ZZZ, B51J0ZA, B51J0ZZ, B51J1ZA, B51J1ZZ, B51JYZA, B51JYZZ, B51JZZA, B51JZZZ, B51K0ZA, B51K0ZZ, B51K1ZA, B51K1ZZ, B51KYZA, B51KYZZ, B51KZZA, B51KZZZ, B51L0ZA, B51L0ZZ, B51L1ZA, B51L1ZZ, B51LYZA, B51LYZZ, B51LZZA, B51LZZZ

© 2022 Ganguli I et al. *JAMA Network Open.*

eTable 5. Procedure Codes for Cascade Procedures

	Event type	Codes (CPT and ICD-10)
**Mammary**	Fine needle aspiration/core breast biopsy	19000-1, 10021-2, 10004-12, 19100-3, 19081-6,
	Lumpectomy/ mastectomy	19126, 19301-2, 19303-7
	Excisional breast biopsy	19120, 19125, 19101
	Breast imaging localization	19281-8
	Lymph node biopsy/excision	38740, 38745, 38525, 38530, 38500, 38505
	Breast repair/reconstruction	19350, 19357, 19361, 19364, 19366, 19367, 19368, 19369, S2066, S2067, S2068,
	Breast radiation	77014, 77371-3, 77385-7, 77401-4, 77406-9, 77411-4, 77416, 77418, 77421-5, 77469, 77520, 77522-3, 77525, 77750, 77761-3, 77767-8, 77770-2, 77776-8, 77785-7, 77789, 79200, 79300, 79403, 79440, 79445, G6002-17, Q3001,<br>0HHT01Z, 0HHT31Z, 0HHT71Z, 0HHT81Z, 0HHTX1Z, 0HHU01Z, 0HHU31Z, 0HHU71Z, 0HHU81Z, 0HHUX1Z, 0HHV01Z, 0HHV31Z, 0HHV71Z, 0HHV81Z, 0HHVX1Z, 0HHW01Z, 0HHW31Z, 0HHW71Z, 0HHW81Z, 0HHWX1Z, 0HHX01Z, 0HHX31Z, 0HHX71Z, 0HHX81Z, 0HHXX1Z, DM000ZZ, DM001ZZ, DM002ZZ, DM003Z0, DM003ZZ, DM004ZZ, DM005ZZ, DM010ZZ, DM011ZZ, DM012ZZ, DM013Z0, DM013ZZ, DM014ZZ, DM015ZZ, DM1097Z, DM1098Z, DM1099Z, DM109BZ, DM109CZ, DM109YZ, DM10B7Z, DM10B8Z, DM10B9Z, DM10BBZ, DM10BCZ, DM10BYZ, DM1197Z, DM1198Z, DM1199Z, DM119BZ, DM119CZ, DM119YZ, DM11B7Z, DM11B8Z, DM11B9Z, DM11BBZ, DM11BCZ, DM11BYZ, DMY07ZZ, DMY0FZZ, DMY17ZZ, DMY1FZZ
**Extramammary**	Liver biopsy, excision, and resection	47000-1, 47100, 47379, 47700<br>0FB00ZX, 0FB00ZZ, 0FB03ZX, 0FB03ZZ, 0FB04ZX, 0FB04ZZ, 0FB10ZX, 0FB10ZZ, 0FB13ZX, 0FB13ZZ, 0FB14ZX, 0FB14ZZ, 0FB20ZX, 0FB20ZZ, 0FB23ZX, 0FB23ZZ, 0FB24ZX, 0FB24ZZ, 0FT00ZZ, 0FT04ZZ, 0FT10ZZ, 0FT14ZZ, 0FT20ZZ, 0FT24ZZ
	Cholecystectomy	47562-4, 47600, 47605, 47610, 47612, 47620,<br>0FT40ZZ, 0FT44ZZ
	Lung biopsy, excision and resection of lungs, pleura, and diaphragm	32400, 32405, 0BBC0ZX, 0BBC0ZZ, 0BBC3ZX, 0BBC3ZZ, 0BBC4ZX, 0BBC4ZZ, 0BBC7ZX, 0BBC7ZZ, 0BBC8ZX, 0BBC8ZZ, 0BBD0ZX, 0BBD0ZZ, 0BBD3ZX, 0BBD3ZZ, 0BBD4ZX, 0BBD4ZZ, 0BBD7ZX, 0BBD7ZZ, 0BBD8ZX, 0BBD8ZZ, 0BBF0ZX, 0BBF0ZZ, 0BBF3ZX, 0BBF3ZZ, 0BBF4ZX, 0BBF4ZZ, 0BBF7ZX, 0BBF7ZZ, 0BBF8ZX, 0BBF8ZZ, 0BBG0ZX, 0BBG0ZZ, 0BBG3ZX, 0BBG3ZZ, 0BBG4ZX, 0BBG4ZZ, 0BBG7ZX, 0BBG7ZZ, 0BBG8ZX, 0BBG8ZZ, 0BBJ0ZX, 0BBJ0ZZ, 0BBJ3ZX, 0BBJ3ZZ, 0BBJ4ZX, 0BBJ4ZZ, 0BBJ7ZX, 0BBJ7ZZ, 0BBJ8ZX, 0BBJ8ZZ , 0BBH0ZX, 0BBH0ZZ, 0BBH3ZX, 0BBH3ZZ, 0BBH4ZX, 0BBH4ZZ, 0BBH7ZX, 0BBH7ZZ, 0BBH8ZX, 0BBH8ZZ, 0BBK0ZX, 0BBK0ZZ, 0BBK3ZX, 0BBK3ZZ, 0BBK4ZX, 0BBK4ZZ, 0BBK7ZX, 0BBK7ZZ, 0BBK8ZX, 0BBK8ZZ, 0BBL0ZX, 0BBL0ZZ, 0BBL3ZX, 0BBL3ZZ, 0BBL4ZX, 0BBL4ZZ, 0BBL7ZX, 0BBL7ZZ, 0BBL8ZX, 0BBL8ZZ, 0BBM0ZX, 0BBM0ZZ, 0BBM3ZX, 0BBM3ZZ, 0BBM4ZX, 0BBM4ZZ, 0BBM7ZX, 0BBM7ZZ, 0BBM8ZX, 0BBM8ZZ , 0BB30ZX, 0BB30ZZ, 0BB33ZX, 0BB33ZZ, 0BB34ZX, 0BB34ZZ, 0BB37ZX, 0BB37ZZ, 0BB38ZX, 0BB38ZZ,

© 2022 Ganguli I et al. *JAMA Network Open.*

	Event type	Codes (CPT and ICD-10)
		0BB70ZX, 0BB70ZZ, 0BB73ZX, 0BB73ZZ, 0BB74ZX, 0BB74ZZ, 0BB77ZX, 0BB77ZZ, 0BB78ZX, 0BB78ZZ , 0BB40ZX, 0BB40ZZ, 0BB43ZX, 0BB43ZZ, 0BB44ZX, 0BB44ZZ, 0BB47ZX, 0BB47ZZ, 0BB48ZX, 0BB48ZZ, 0BB50ZX, 0BB50ZZ, 0BB53ZX, 0BB53ZZ, 0BB54ZX, 0BB54ZZ, 0BB57ZX, 0BB57ZZ, 0BB58ZX, 0BB58ZZ, 0BB60ZX, 0BB60ZZ, 0BB63ZX, 0BB63ZZ, 0BB64ZX, 0BB64ZZ, 0BB67ZX, 0BB67ZZ, 0BB68ZX, 0BB68ZZ, 0BB80ZX, 0BB80ZZ, 0BB83ZX, 0BB83ZZ, 0BB84ZX, 0BB84ZZ, 0BB87ZX, 0BB87ZZ, 0BB88ZX, 0BB88ZZ, 0BBB0ZX, 0BBB0ZZ, 0BBB3ZX, 0BBB3ZZ, 0BBB4ZX, 0BBB4ZZ, 0BBB7ZX, 0BBB7ZZ, 0BBB8ZX, 0BBB8ZZ, 0BB90ZX, 0BB90ZZ, 0BB93ZX, 0BB93ZZ, 0BB94ZX, 0BB94ZZ, 0BB97ZX, 0BB97ZZ, 0BB98ZX, 0BB98ZZ , 0BBN0ZX, 0BBN0ZZ, 0BBN3ZX, 0BBN3ZZ, 0BBN4ZX, 0BBN4ZZ, 0BBN8ZX, 0BBN8ZZ, 0BBP0ZX, 0BBP0ZZ, 0BBP3ZX, 0BBP3ZZ, 0BBP4ZX, 0BBP4ZZ, 0BBP8ZX, 0BBP8ZZ, 0BBT0ZX, 0BBT0ZZ, 0BBT3ZX, 0BBT3ZZ, 0BBT4ZX, 0BBT4ZZ, 0BTC0ZZ, 0BTC4ZZ, 0BTD0ZZ, 0BTD4ZZ, 0BTF0ZZ, 0BTF4ZZ, 0BTG0ZZ, 0BTG4ZZ, 0BTJ0ZZ, 0BTJ4ZZ, 0BTK0ZZ, 0BTK4ZZ, 0BTL0ZZ, 0BTL4ZZ, 0BTM0ZZ, 0BTM4ZZ, 0BTH0ZZ, 0BTH4ZZ, 0BT40ZZ, 0BT44ZZ, 0BT50ZZ, 0BT54ZZ, 0BT60ZZ, 0BT64ZZ, 0BT80ZZ, 0BT84ZZ, 0BTB0ZZ, 0BTB4ZZ , 0BT30ZZ, 0BT34ZZ, 0BT70ZZ, 0BT74ZZ, 0BT90ZZ, 0BT94ZZ , 0BTT0ZZ, 0BTT4ZZ
	Thoracotomy	32100, 32110, 32120, 32124, 32140, 32141, 32150, 32151, 32160, 32505, 32506, 32507, 32096, 32097, 32098,
	Thorascopy, excision of heart, resection of heart, extirpation of matter from thorax	32601, 32604, 32606, 32607, 32608, 32609, 32650, 32651, 32652, 32653, 32654, 32655, 32656, 32658, 32659, 32661, 32662, 32663, 32664, 32665, 32666, 32667, 32668, 32669, 32670, 32671, 32672, 32673, 32674, 0WJ93ZZ, 0WJ94ZZ, 0WJB0ZZ, 0WJB3ZZ, 0WJB4ZZ, 0WJC0ZZ, 0WJC3ZZ, 0WJC4ZZ, 0WJD0ZZ, 0WJD3ZZ, 0WJD4ZZ, 0WJ80ZZ, 0WJ83ZZ, 0WJ84ZZ, 0WJ8XZZ, 02JA0ZZ, 02JA3ZZ, 02JA4ZZ, 02JY0ZZ, 02JY3ZZ, 02JY4ZZ, 0BJ00ZZ, 0BJ03ZZ, 0BJ04ZZ, 0BJ07ZZ, 0BJ08ZZ, 0BJ0XZZ, 0BJ10ZZ, 0BJ13ZZ, 0BJ14ZZ, 0BJ17ZZ, 0BJ18ZZ, 0BJ1XZZ , 0BJK0ZZ, 0BJK3ZZ, 0BJK4ZZ, 0BJK7ZZ, 0BJK8ZZ, 0BJKXZZ, 0BJL0ZZ, 0BJL3ZZ, 0BJL4ZZ, 0BJL7ZZ, 0BJL8ZZ, 0BJLXZZ, 0BJQ0ZZ, 0BJQ3ZZ, 0BJQ4ZZ, 0BJQ7ZZ, 0BJQ8ZZ, 0BJQXZZ, 0BJT0ZZ, 0BJT3ZZ, 0BJT4ZZ, 0BJT7ZZ, 0BJT8ZZ, 0BJTXZZ<br>02B40ZX, 02B40ZZ, 02B43ZX, 02B43ZZ, 02B44ZX, 02B44ZZ, 02B50ZX, 02B50ZZ, 02B53ZX, 02B53ZZ, 02B54ZX, 02B54ZZ, 02B60ZX, 02B60ZZ, 02B63ZX, 02B63ZZ, 02B64ZX, 02B64ZZ, 02B70ZK, 02B70ZX, 02B70ZZ, 02B73ZK, 02B73ZX, 02B73ZZ, 02B74ZK, 02B74ZX, 02B74ZZ, 02B80ZX, 02B80ZZ, 02B83ZX, 02B83ZZ, 02B84ZX, 02B84ZZ, 02B90ZX, 02B90ZZ, 02B93ZX, 02B93ZZ, 02B94ZX, 02B94ZZ, 02BD0ZX, 02BD0ZZ, 02BD3ZX, 02BD3ZZ, 02BD4ZX, 02BD4ZZ, 02BF0ZX, 02BF0ZZ, 02BF3ZX, 02BF3ZZ, 02BF4ZX, 02BF4ZZ, 02BG0ZX, 02BG0ZZ, 02BG3ZX, 02BG3ZZ, 02BG4ZX, 02BG4ZZ, 02BH0ZX, 02BH0ZZ, 02BH3ZX, 02BH3ZZ, 02BH4ZX, 02BH4ZZ, 02BJ0ZX, 02BJ0ZZ, 02BJ3ZX, 02BJ3ZZ, 02BJ4ZX, 02BJ4ZZ, 02BK0ZX, 02BK0ZZ, 02BK3ZX, 02BK3ZZ, 02BK4ZX, 02BK4ZZ, 02BL0ZX, 02BL0ZZ, 02BL3ZX, 02BL3ZZ, 02BL4ZX, 02BL4ZZ, 02BM0ZX, 02BM0ZZ, 02BM3ZX, 02BM3ZZ, 02BM4ZX, 02BM4ZZ, 02BN0ZX, 02BN0ZZ, 02BN3ZX, 02BN3ZZ, 02BN4ZX, 02BN4ZZ, 02BP0ZX, 02BP0ZZ, 02BP3ZX, 02BP3ZZ, 02BP4ZX, 02BP4ZZ, 02BQ0ZX, 02BQ0ZZ, 02BQ3ZX,

© 2022 Ganguli I et al. *JAMA Network Open.*

	Event type	Codes (CPT and ICD-10)
		02BQ3ZZ, 02BQ4ZX, 02BQ4ZZ, 02BR0ZX, 02BR0ZZ, 02BR3ZX, 02BR3ZZ, 02BR4ZX, 02BR4ZZ, 02BS0ZX, 02BS0ZZ, 02BS3ZX, 02BS3ZZ, 02BS4ZX, 02BS4ZZ, 02BT0ZX, 02BT0ZZ, 02BT3ZX, 02BT3ZZ, 02BT4ZX, 02BT4ZZ, 02BV0ZX, 02BV0ZZ, 02BV3ZX, 02BV3ZZ, 02BV4ZX, 02BV4ZZ, 02BW0ZX, 02BW0ZZ, 02BW3ZX, 02BW3ZZ, 02BW4ZX, 02BW4ZZ, 02BX0ZX, 02BX0ZZ, 02BX3ZX, 02BX3ZZ, 02BX4ZX, 02BX4ZZ,<br>02T50ZZ, 02T53ZZ, 02T54ZZ, 02T80ZZ, 02T83ZZ, 02T84ZZ, 02T90ZZ, 02T93ZZ, 02T94ZZ, 02TD0ZZ, 02TD3ZZ, 02TD4ZZ, 02TH0ZZ, 02TH3ZZ, 02TH4ZZ, 02TM0ZZ, 02TM3ZZ, 02TM4ZZ, 02TN0ZZ, 02TN3ZZ, 02TN4ZZ,<br>0BC30ZZ, 0BC33ZZ, 0BC34ZZ, 0BC37ZZ, 0BC38ZZ, 0BC40ZZ, 0BC43ZZ, 0BC44ZZ, 0BC47ZZ, 0BC48ZZ, 0BC50ZZ, 0BC53ZZ, 0BC54ZZ, 0BC57ZZ, 0BC58ZZ, 0BC60ZZ, 0BC63ZZ, 0BC64ZZ, 0BC67ZZ, 0BC68ZZ, 0BC70ZZ, 0BC73ZZ, 0BC74ZZ, 0BC77ZZ, 0BC78ZZ, 0BC80ZZ, 0BC83ZZ, 0BC84ZZ, 0BC87ZZ, 0BC88ZZ, 0BC90ZZ, 0BC93ZZ, 0BC94ZZ, 0BC97ZZ, 0BC98ZZ, 0BCB0ZZ, 0BCB3ZZ, 0BCB4ZZ, 0BCB7ZZ, 0BCB8ZZ, 0BCC0ZZ, 0BCC3ZZ, 0BCC4ZZ, 0BCC7ZZ, 0BCC8ZZ, 0BCD0ZZ, 0BCD3ZZ, 0BCD4ZZ, 0BCD7ZZ, 0BCD8ZZ, 0BCF0ZZ, 0BCF3ZZ, 0BCF4ZZ, 0BCF7ZZ, 0BCF8ZZ, 0BCG0ZZ, 0BCG3ZZ, 0BCG4ZZ, 0BCG7ZZ, 0BCG8ZZ, 0BCH0ZZ, 0BCH3ZZ, 0BCH4ZZ, 0BCH7ZZ, 0BCH8ZZ, 0BCJ0ZZ, 0BCJ3ZZ, 0BCJ4ZZ, 0BCJ7ZZ, 0BCJ8ZZ, 0BCK0ZZ, 0BCK3ZZ, 0BCK4ZZ, 0BCK7ZZ, 0BCK8ZZ, 0BCL0ZZ, 0BCL3ZZ, 0BCL4ZZ, 0BCL7ZZ, 0BCL8ZZ, 0BCM0ZZ, 0BCM3ZZ, 0BCM4ZZ, 0BCM7ZZ, 0BCM8ZZ, 0BCN0ZZ, 0BCN3ZZ, 0BCN4ZZ, 0BCP0ZZ, 0BCP3ZZ, 0BCP4ZZ, 0BCT0ZZ, 0BCT3ZZ, 0BCT4ZZ, 02C00Z6, 02C00ZZ, 02C03Z6, 02C03ZZ, 02C04Z6, 02C04ZZ, 02C10Z6, 02C10ZZ, 02C13Z6, 02C13ZZ, 02C14Z6, 02C14ZZ, 02C20Z6, 02C20ZZ, 02C23Z6, 02C23ZZ, 02C24Z6, 02C24ZZ, 02C30Z6, 02C30ZZ, 02C33Z6, 02C33ZZ, 02C34Z6, 02C34ZZ, 02C40ZZ, 02C43ZZ, 02C44ZZ, 02C50ZZ, 02C53ZZ, 02C54ZZ, 02C60ZZ, 02C63ZZ, 02C64ZZ, 02C70ZZ, 02C73ZZ, 02C74ZZ, 02C80ZZ, 02C83ZZ, 02C84ZZ, 02C90ZZ, 02C93ZZ, 02C94ZZ, 02CD0ZZ, 02CD3ZZ, 02CD4ZZ, 02CF0ZZ, 02CF3ZZ, 02CF4ZZ, 02CG0ZZ, 02CG3ZZ, 02CG4ZZ, 02CH0ZZ, 02CH3ZZ, 02CH4ZZ, 02CJ0ZZ, 02CJ3ZZ, 02CJ4ZZ, 02CK0ZZ, 02CK3ZZ, 02CK4ZZ, 02CL0ZZ, 02CL3ZZ, 02CL4ZZ, 02CM0ZZ, 02CM3ZZ, 02CM4ZZ, 02CN0ZZ, 02CN3ZZ, 02CN4ZZ, 02CP0ZZ, 02CP3ZZ, 02CP4ZZ, 02CQ0ZZ, 02CQ3ZZ, 02CQ4ZZ, 02CR0ZZ, 02CR3ZZ, 02CR4ZZ, 02CS0ZZ, 02CS3ZZ, 02CS4ZZ, 02CT0ZZ, 02CT3ZZ, 02CT4ZZ, 02CV0ZZ, 02CV3ZZ, 02CV4ZZ, 02CW0ZZ, 02CW3ZZ, 02CW4ZZ, 02CX0ZZ, 02CX3ZZ, 02CX4ZZ
	Mediastinoscopy	39000, 39010, 39401, 39402, 0WJC4ZZ
	Bronchoscopy	31622-38, 31640-1, 31643, 31645-9, 31651-4 31660-1,
	Transthoracic echocardiogram, including Doppler	93303, 93304, 93306, 93307, 93308, 93350, 93351, 93352, 0439T, 93320-1, 93325<br>B244ZZZ, B244ZZ3, B244YZZ , B245YZZ, B245ZZZ, B245ZZ3, B246YZZ, B246ZZ3, B246ZZZ, B24BYZZ, B24BZZ3, B24BZZZ
	Transesophageal echocardiogram	93312, 93313, 93314, 93315, 93316, 93317, 93318, 93355, B244ZZ4, B245ZZ4, B246ZZ4, B24BZZ4, B24CZZ4, B240ZZ4, B241ZZ4
	Thyroid cyst aspiration	60300, 10022

© 2022 Ganguli I et al. *JAMA Network Open.*

	Event type	Codes (CPT and ICD-10)
	**Event type**	**Codes (CPT and ICD-10)**
	Thyroid biopsy, excision	60100, 76940, 76942, 77012, 77021<br>0GBG3ZX, 0GBH3ZX, 0GBJ3ZX,
	Thyroidectomy	60210, 60212, 60220, 60225, 60240, 60252, 60254, 60260, 60270, 60271,<br>0GTG0ZZ, 0GTG4ZZ, 0GTH0ZZ, 0GTH4ZZ, 0GTJ0ZZ, 0GTJ4ZZ, 0GTK0ZZ, 0GTK4ZZ
	Retroperitoneal, Renal biopsy	49180, 50200, 50205<br>0WBH0ZX, 0WBH0ZZ, 0WBH3ZX, 0WBH3ZZ, 0WBH4ZX, 0WBH4ZZ, 0TB00ZX, 0TB00ZZ, 0TB03ZX, 0TB03ZZ, 0TB04ZX, 0TB04ZZ, 0TB07ZX, 0TB07ZZ, 0TB08ZX, 0TB08ZZ, 0TB10ZX, 0TB10ZZ, 0TB13ZX, 0TB13ZZ, 0TB14ZX, 0TB14ZZ, 0TB17ZX, 0TB17ZZ, 0TB18ZX, 0TB18ZZ, 0TB18ZZ, 0TB30ZZ, 0TB33ZX, 0TB33ZZ, 0TB34ZX, 0TB34ZZ, 0TB37ZX, 0TB37ZZ, 0TB38ZX, 0TB38ZZ, 0TB40ZX, 0TB40ZZ, 0TB43ZX, 0TB43ZZ, 0TB44ZX, 0TB44ZZ, 0TB47ZX, 0TB47ZZ, 0TB48ZX, 0TB48ZZ
	Nephrectomy, resection of kidneys, ureter, bladder	50543, 50545, 50548, 50220, 50225, 50230, 50234, 50236, 50240<br>0TT00ZZ, 0TT04ZZ, 0TT10ZZ, 0TT14ZZ, 0TT20ZZ, 0TT24ZZ, 0TT30ZZ, 0TT34ZZ, 0TT37ZZ, 0TT38ZZ, 0TT40ZZ, 0TT44ZZ, 0TT47ZZ, 0TT48ZZ, 0TT60ZZ, 0TT64ZZ, 0TT67ZZ, 0TT68ZZ, 0TT70ZZ, 0TT74ZZ, 0TT77ZZ, 0TT78ZZ, 0TTB0ZZ, 0TTB4ZZ, 0TTB7ZZ, 0TTB8ZZ, 0TTC0ZZ, 0TTC4ZZ, 0TTC7ZZ, 0TTC8ZZ, 0TTD0ZZ, 0TTD4ZZ, 0TTD7ZZ, 0TTD8ZZ
	Renal laparoscopy	50541, 50542, 50543, 50544, 50545, 50546, 50547, 50548, 50549
	Lithotripsy	50590<br>0TF30ZZ, 0TF33ZZ, 0TF34ZZ, 0TF37ZZ, 0TF38ZZ, 0TF3XZZ, 0TF40ZZ, 0TF43ZZ, 0TF44ZZ, 0TF47ZZ, 0TF48ZZ, 0TF4XZZ, 0TF60ZZ, 0TF63ZZ, 0TF64ZZ, 0TF67ZZ, 0TF68ZZ, 0TF6XZZ, 0TF70ZZ, 0TF73ZZ, 0TF74ZZ, 0TF77ZZ, 0TF78ZZ, 0TF7XZZ, 0TFB0ZZ, 0TFB3ZZ, 0TFB4ZZ, 0TFB7ZZ, 0TFB8ZZ, 0TFBXZZ, 0TFC0ZZ, 0TFC3ZZ, 0TFC4ZZ, 0TFC7ZZ, 0TFC8ZZ, 0TFCXZZ, 0TFD0ZZ, 0TFD3ZZ, 0TFD4ZZ, 0TFD7ZZ, 0TFD8ZZ, 0TFDXZZ
	Litholapaxy	52317-8<br>0TCB0ZZ, 0TCB3ZZ, 0TCB4ZZ, 0TCB7ZZ, 0TCB8ZZ
	Renal ablation	50592-3, 50250<br>0T500ZZ, 0T503ZZ, 0T504ZZ, 0T507ZZ, 0T508ZZ, 0T510ZZ, 0T513ZZ, 0T514ZZ, 0T517ZZ, 0T518ZZ
	Cystourethroscopy	52000-1, 52005, 52007, 52010, 52204, 52214, 52224, 52234-5, 52240, 52250, 52260, 52265, 52270, 52276-7, 52281-3, 52285, 52287, 52290, 52300, 52301, 52305, 52310, 52315, 52320, 52325, 52327, 52330, 52332, 52334, 52341-6, 52351-6
	Magnetic resonance cholangiopancreatography	S8037

© 2022 Ganguli I et al. *JAMA Network Open.*

eTable 6. Procedure codes for cascade visits and Specialty Codes for Cascase Specialty Visits

A. Procedure Codes for Cascade Visits

Visits were defined based on the following HCPCS codes:

**Codes (HCPCS)**	99201, 99202, 99203, 99204, 99205, 99206, 99207, 99208, 99209, 99210, 99211, 99212, 99213, 99214, 99215, 99381, 99382, 99383, 99384, 99385, 99386, 99387, 99391, 99392, 99393, 99394, 99395, 99396, 99397, G0463

We analyzed visits with specialists relevant to mammary and extramammary findings, respectively:

B. Specialty codes for cascade specialty visits

	**Specialties**	**Specialty codes**
**Mammary**	Oncology (including medical, radiation), General surgery, Internal medicine, Preventive medicine, NP, PA	380, 500, 505, 204, 360, 825, 845
**Extramammary**	General surgery, Colorectal surgery, Cardiothoracic surgery, Vascular surgery, Gastroenterology, Cardiology, Pulmonology, Rheumatology, Orthopedics, Oncology (including medical, radiation), Hematology, Infectious disease, Endocrinology, Nephrology, Internal medicine, Preventive medicine, NP, PA	500, 505, 510, 535, 540, 580, 585, 550, 275, 250, 295, 300, 530, 380, 280, 285, 270, 290, 204, 360, 825, 845

© 2022 Ganguli I et al. *JAMA Network Open.*

eTable 7. Diagnosis Codes for Cascade New Diagnoses[a]

Organ/category	ICD-10 codes
**Mammary**	
Breast	C50.XXX, D05.1X, C79.81, N60.8X, N60.9X, D05.0X, D48.6X, D24.X, N60.0X, N60.1X, N60.2X, N60.3X, N60.4X, N62, D05.8X, D05.9X, R92.0, R92.1, R92.8, N64.1, N64.59
**Extramammary**	
Liver	K70.0, K70.10, K70.11, K70.2, K70.30, K70.31, K70.40, K70.41, K70.9, K71.0, K71.11, K71.7, K71.8, K71.9, K72.00, K72.01, K72.10, K72.11, K72.90, K72.91, K74.0, K74.1, K74.2, K74.3, K74.4, K74.5, K74.60, K74.69, K75.0, K75.1, K75.81, K75.89, K75.9, K76.0, K76.1, K76.2, K76.3, K76.5, K76.6, K76.7, K76.81, K76.89, K76.9, K77, R16.0, R16.2, D13.4 , C22, C22.0, C22.1, C22.2, C22.3, C22.4, C22.7, C22.8, C22.9, C78.7, B15.0, B15.9, B16.0, B16.1, B16.2, B16.9, B17.0, B17.10, B17.11, B17.2, B17.8, B17.9, B18.0, B18.1, B18.2, B18.8, B18.9, B19.0, B19.10, B19.11, B19.20, B19.21, B19.9, Q44, Q44.0, Q44.1, Q44.2, Q44.3, Q44.4, Q44.5, Q44.6, Q44.7, D18.03, B67.8, R93.2
Gallbladder	K80, K80.0, K80.00, K80.01, K80.1, K80.10, K80.11, K80.12, K80.13, K80.18, K80.19, K80.2, K80.20, K80.21, K80.3, K80.30, K80.31, K80.32, K80.33, K80.34, K80.35, K80.36, K80.37, K80.4, K80.40, K80.41, K80.42, K80.43, K80.44, K80.45, K80.46, K80.47, K80.5, K80.50, K80.51, K80.6, K80.60, K80.61, K80.62, K80.63, K80.64, K80.65, K80.66, K80.67, K80.7, K80.70, K80.71, K80.8, K80.80, K80.81, K82, K82.0, K82.1, K82.2, K82.3, K82.4, K82.8, K82.9, K82.A, K82.A1, K82.A2, K87, C23, C24.8, C24.9, S36.1 , S36.11, S36.112, S36.112A, S36.112D, S36.112S, S36.113, S36.113A, S36.113D, S36.113S, S36.114, S36.114A, S36.114D, S36.114S, S36.115, S36.115A, S36.115D, S36.115S, S36.116, S36.116A, S36.116D, S36.116S, S36.118, S36.118A, S36.118D, S36.118S, S36.119, S36.119A, S36.119D, S36.119S, S36.12, S36.122, S36.122A, S36.122D, S36.122S, S36.123, S36.123A, S36.123D, S36.123S, S36.128, S36.128A, S36.128D, S36.128S, S36.129, S36.129A, S36.13, S36.13XA, S36.13XD, S36.13XS, D37.6, D13.5, D01.5 , K83, K83.0, K83.01, K83.09, K83.1, K83.2, K83.3, K83.4, K83.5, K83.8, K83.9, O26.6, O26.61, O26.611, O26.612, O26.613, O26.619, O26.62, O26.63
Pancreas	K86.0, K86.1, K86.2, K86.3, K86.8, K86.81, K86.89, K86.9, K85, K85.0, K85.00, K85.01, K85.02, K85.1, K85.10, K85.11, K85.12, K85.2, K85.20, K85.21, K85.22, K85.3, K85.30, K85.31, K85.32, K85.8, K85.80, K85.81, K85.82, K85.9, K85.90, K85.92, C25, C25.0, C25.1, C25.2, C25.3, C25.4, C25.7, C25.8, C25.9, D13.6 , D13.7 , D37.8, D01.7, S36.2, S36.20, S36.200, S36.200A, S36.200D, S36.200S, S36.201, S36.201A, S36.201D, S36.201S, S36.202, S36.202A, S36.202D, S36.202S, S36.209, S36.209A, S36.209D, S36.209S, S36.22, S36.220, S36.220A, S36.220D, S36.220S, S36.221, S36.221A, S36.221D, S36.221S, S36.222, S36.222A, S36.222D, S36.222S, S36.229, S36.229A, S36.229D, S36.229S, S36.23, S36.230, S36.230A, S36.230D, S36.230S, S36.231, S36.231A, S36.231D, S36.231S, S36.232, S36.232A, S36.232D, S36.232S, S36.239, S36.239A, S36.239D, S36.239S, S36.24, S36.240, S36.240A, S36.240D, S36.240S, S36.241, S36.241A, S36.241D, S36.241S, S36.242, S36.242A, S36.242D, S36.242S, S36.249, S36.249A, S36.249D, S36.249S, S36.25, S36.250, S36.250A, S36.250D, S36.250S, S36.251, S36.251A, S36.251D, S36.251S, S36.252, S36.252A, S36.252D, S36.252S, S36.259, S36.259A, S36.259D, S36.259S, S36.26, S36.260, S36.260A, S36.260D, S36.260S, S36.261, S36.261A, S36.261D, S36.261S, S36.262, S36.262A, S36.262D, S36.262S, S36.269, S36.269A, S36.269D, S36.269S, S36.29, S36.290, S36.290A, S36.290D, S36.290S, S36.291, S36.291A, S36.291D, S36.291S, S36.292, S36.292A, S36.292D, S36.292S, S36.299, S36.299A, S36.299D, S36.299S
Thymus	C37, D15.0, E32.1

© 2022 Ganguli I et al. *JAMA Network Open.*

Organ/category	ICD-10 codes
Lungs	J90, J91.0, J91, J91.8, J94.0 , R91, R91.1, R91.8, J15, J15.0, J15.1, J15.2, J15.20, J15.21, J15.211, J15.212, J15.29, J15.3, J15.4, J15.5, J15.6, J15.7, J15.8, J15.9, J16, J16.0, J16.8, J12, J12.0, J12.1, J12.2, J12.3, J12.8, J12.81, J12.82, J12.89, J12.9, J13, J84.2, J17, J85, J85.0, J85.1, J85.2, J85.3, J98.1, J98.11, J98.19, Q33.0, J98.4, C34, C34.0, C34.00, C34.01, C34.02, C34.1, C34.10, C34.11, C34.12, C34.2, C34.3, C34.30, C34.31, C34.32, C34.8, C34.80, C34.81, C34.82, C34.9, C34.90, C34.91, C34.92, J40, J41.0, J41.1, J41.8, J42, J43.0, J43.1, J43.2, J43.8, J43.9, J44.0, J44.1, J44.9, J47.0, J47.1, J47.9
Heart	I51.3 , I31.8, I31.3, I30.
Musculoskeletal	S22.3, S22.31, S22.31XA, S22.31XB, S22.31XD, S22.31XG, S22.31XK, S22.31XS, S22.32, S22.32XA, S22.32XB, S22.32XD, S22.32XG, S22.32XK, S22.32XS, S22.39, S22.39XA, S22.39XB, S22.39XD, S22.39XG, S22.39XK, S22.39XS, S22.4, S22.41, S22.41XA, S22.41XB, S22.41XD, S22.41XG, S22.41XK, S22.41XS, S22.42, S22.42XA, S22.42XB, S22.42XD, S22.42XG, S22.42XK, S22.42XS, S22.43, S22.43XA, S22.43XB, S22.43XD, S22.43XG, S22.43XK, S22.43XS, S22.49, S22.49XA, S22.49XB, S22.49XD, S22.49XG, S22.49XK, S22.49XS, S42.0, S42.00, S42.001, S42.001A, S42.001B, S42.001D, S42.001G, S42.001K, S42.001P, S42.001S, S42.002, S42.002A, S42.002B, S42.002D, S42.002G, S42.002K, S42.002P, S42.002S, S42.009, S42.009A, S42.009B, S42.009D, S42.009G, S42.009K, S42.009P, S42.009S, S42.01, S42.011, S42.011A, S42.011B, S42.011D, S42.011G, S42.011K, S42.011P, S42.011S, S42.012, S42.012A, S42.012B, S42.012D, S42.012G, S42.012K, S42.012P, S42.012S, S42.013, S42.013A, S42.013B, S42.013D, S42.013G, S42.013K, S42.013P, S42.013S, S42.014, S42.014A, S42.014B, S42.014D, S42.014G, S42.014K, S42.014P, S42.014S, S42.015, S42.015A, S42.015B, S42.015D, S42.015G, S42.015K, S42.015P, S42.015S, S42.016, S42.016A, S42.016B, S42.016D, S42.016G, S42.016K, S42.016P, S42.016S, S42.017, S42.017A, S42.017B, S42.017D, S42.017G, S42.017K, S42.017P, S42.017S, S42.018, S42.018A, S42.018B, S42.018D, S42.018G, S42.018K, S42.018P, S42.018S, S42.019, S42.019A, S42.019B, S42.019D, S42.019G, S42.019K, S42.019P, S42.019S, S42.02, S42.021, S42.021A, S42.021B, S42.021D, S42.021G, S42.021K, S42.021P, S42.021S, S42.022, S42.022A, S42.022B, S42.022D, S42.022G, S42.022K, S42.022P, S42.022S, S42.023, S42.023A, S42.023B, S42.023D, S42.023G, S42.023K, S42.023P, S42.023S, S42.024, S42.024A, S42.024B, S42.024D, S42.024G, S42.024K, S42.024P, S42.024S, S42.025, S42.025A, S42.025B, S42.025D, S42.025G, S42.025K, S42.025P, S42.025S, S42.026, S42.026A, S42.026B, S42.026D, S42.026G, S42.026K, S42.026P, S42.026S, S42.03, S42.031, S42.031A, S42.031B, S42.031D, S42.031G, S42.031K, S42.031P, S42.031S, S42.032, S42.032A, S42.032B, S42.032D, S42.032G, S42.032K, S42.032P, S42.032S, S42.033, S42.033A, S42.033B, S42.033D, S42.033G, S42.033K, S42.033P, S42.033S, S42.034, S42.034A, S42.034B, S42.034D, S42.034G, S42.034K, S42.034P, S42.034S, S42.035, S42.035A, S42.035B, S42.035D, S42.035G, S42.035K, S42.035P, S42.035S, S42.036, S42.036A, S42.036B, S42.036D, S42.036G, S42.036K, S42.036P, S42.036S, S42.9, S42.90, S42.90XA, S42.90XB, S42.90XD, S42.90XG, S42.90XK, S42.90XP, S42.90XS, S42.91, S42.91XA, S42.91XB, S42.91XD, S42.91XG, S42.91XK, S42.91XP, S42.91XS, S42.92, S42.92XA, S42.92XB, S42.92XD, S42.92XG, S42.92XK, S42.92XP, S42.92XS, C41.3, C41.2 , C41.9 , C79.5, C79.51, C79.52, M62.18, M62.11, M62.111, M62.112, M62.119, M62.10, D18.09, M89.9 , M51, M51.0, M51.04, M51.05, M51.06, M51.1, M51.14, M51.15, M51.16, M51.17, M51.2, M51.24, M51.25, M51.26, M51.27, M51.3, M51.34, M51.35, M51.36, M51.37, M51.4, M51.44, M51.45, M51.46, M51.47, M51.8, M51.84, M51.85, M51.86, M51.87, M51.
Mediastinal/axillary lymph nodes	R59, R59.0, R59.1, R59.9, C77.3, D36.0
Vascular	D18.0, D18.00, D18.01, D18.02, Q26, Q26.0, Q26.1, Q26.2, Q26.3, Q26.4, Q26.5, Q26.6, Q26.8, Q26.9
Thyroid	C73 , E04, E04.0, E04.1, E04.2, E04.8, E04.9, E07.1, E06.0, E06.1, E06.2, E06.3, E06.4, E06.9, E07.0, E07.8, E07.81, E07.89, E07.9, D34 , E05.0, E05.00, E05.01, E05.1, E05.10, E05.11, E05.2, E05.20, E05.21, E05.3, E05.30, E05.31, E05.4, E05.40, E05.41, E05.8, E05.80, E05.81, E05.9, E05.90, E05.91, E03, E03.0, E03.1, E03.2, E03.3, E03.4, E03.5, E03.8, E03.9, E01.8, E02, E89.0

© 2022 Ganguli I et al. *JAMA Network Open.*

Organ/category	ICD-10 codes
Spleen	D73, D73.0, D73.1, D73.2, D73.3, D73.4, D73.5, D73.8, D73.81, D73.89, D73.9, C26.1 , R16.1, R16.2, Q89.0, Q89.01, Q89.0
Kidney	C64.1, C64.2, C64.9, C68.9, D30.00, D30.01, D30.02, D41.00, D41.01, D41.02, D41.10, D41.11, D41.12, D41.20, D41.21, D41.22, D59.3, E08.21, E08.22, E08.29, E08.65, E09.21, E09.22, E09.29, E10.21, E10.22, E10.29, E10.65, E11.21, E11.22, E11.29, E11.65, E13.21, E13.22, E13.29, E74.8, I12.0, I12.9, I13.0, I13.10, I13.11, I13.2, I70.1, I72.2, K76.7, M10.30, M10.311, M10.312, M10.319, M10.321, M10.322, M10.329, M10.331, M10.332, M10.339, M10.341, M10.342, M10.349, M10.351, M10.352, M10.359, M10.361, M10.362, M10.369, M10.371, M10.372, M10.379, M10.38, M10.39, M32.14, M32.15, M35.04, N00.0, N00.1, N00.2, N00.3, N00.4, N00.5, N00.6, N00.7, N00.8, N00.9, N01.0, N01.1, N01.2, N01.3, N01.4, N01.5, N01.6, N01.7, N01.8, N01.9, N02.0, N02.1, N02.2, N02.3, N02.4, N02.5, N02.6, N02.7, N02.8, N02.9, N03.0, N03.1, N03.2, N03.3, N03.4, N03.5, N03.6, N03.7, N03.8, N03.9, N04.0, N04.1, N04.2, N04.3, N04.4, N04.5, N04.6, N04.7, N04.8, N04.9, N05.0, N05.1, N05.2, N05.3, N05.4, N05.5, N05.6, N05.7, N05.8, N05.9, N06.0, N06.1, N06.2, N06.3, N06.4, N06.5, N06.6, N06.7, N06.8, N06.9, N07.0, N07.1, N07.2, N07.3, N07.4, N07.5, N07.6, N07.7, N07.8, N07.9, N08, N13.1, N13.2, N13.30, N13.39, N14.0, N14.1, N14.2, N14.3, N14.4, N15.0, N15.8, N15.9, N16, N17.0, N17.1, N17.2, N17.8, N17.9, N18.1, N18.2, N18.3, N18.4, N18.5, N18.6, N18.9, N19, N25.0, N25.1, N25.81, N25.89, N25.9, N26.1, N26.9, Q61.11, Q61.4, Q62.0, Q62.2, Q62.31, Q62.32, R94.4 , C79.0, C79.00, C79.01, C79.02, C79.1, C79.10, C79.11, C79.19, N28.1, Q61.00, Q61.9, Q61.3, Q61.2, Q61.19, Q61.5, Q61.02, Q61.8, N20, N20.0, N20.1, N20.2, N20.9, Q61.4, Q62.11, Q62.10, Q62.39, Q62.12, N30, N30.0, N30.00, N30.01, N30.1, N30.10, N30.11, N30.2, N30.20, N30.21, N30.3, N30.30, N30.31, N30.4, N30.40, N30.41, N30.8, N30.80, N30.81, N30.9, N30.90, N30.91, N34, N34.0, N34.1, N34.2, N34.3, N73.9, N12, C65, C65.1, C65.2, C65.9
Adrenal	D35.0, D35.00, D35.01, D35.02, C74, C74.0, C74.00, C74.01, C74.02, C74.1, C74.10, C74.11, C74.12, C74.9, C74.90, C74.91, C74.92, C79.7, C79.70, C79.71, C79.72, E27, E27.0, E27.2, E27.3, E27.4, E27.40, E27.49, E27.5, E27.8, E27.9, Q89.1, S37.81, S37.812, S37.812A, S37.812D, S37.812S, S37.813, S37.813A, S37.813D, S37.813S, S37.818, S37.818A, S37.818D, S37.818S, S37.819, S37.819A, S37.819D, S37.819S, D44.1, D44.10, D44.11, D44.12
GI not otherwise specified	C18, C18.0, C18.1, C18.2, C18.3, C18.4, C18.5, C18.6, C18.7, C18.8, C18.9, C19, C20, D01, D01.0, D01.1, D01.2, D01.3, D01.4, D01.40, D01.49, D01.9, R85, R85.0, R85.1, R85.2, R85.3, R85.4, R85.5, R85.6, R85.61, R85.610, R85.611, R85.612, R85.613, R85.614, R85.615, R85.616, R85.618, R85.619, R85.69, R85.7, R85.8, R85.81, R85.82, R85.89, R85.9, D13, D13.0, D13.1, D13.2, D13.3, D13.30, D13.39, D13.9, K31.9, C49.A, C49.A0, C49.A1, C49.A2, C49.A3, C49.A4, C49.A5, C49.A9, K44, K44.0, K44.1, K44.9, K40, K40.0, K40.00, K40.01, K40.1, K40.10, K40.11, K40.2, K40.20, K40.21, K40.3, K40.30, K40.31, K40.4, K40.40, K40.41, K40.9, K40.90, K40.91
Intraperitoneal	R18, R18.0, R18.8
Infectious (syphilis, tuberculosis, malaria	A52.75, A15, A15.0, A15.4, A15.5, A15.6, A15.7, A15.8, A15.9, A17, A17.0, A17.1, A17.8, A17.81, A17.82, A17.83, A17.89, A17.9, A18, A18.0, A18.01, A18.02, A18.03, A18.09, A18.1, A18.10, A18.11, A18.12, A18.13, A18.14, A18.15, A18.16, A18.17, A18.18, A18.2, A18.3, A18.31, A18.32, A18.39, A18.4, A18.5, A18.50, A18.51, A18.52, A18.53, A18.54, A18.59, A18.6, A18.7, A18.8, A18.81, A18.82, A18.83, A18.84, A18.85, A18.89, A19, A19.1, A19.2, A19.3, A19.8, A19.9, Z22.7, B90, B90.0, B90.1, B90.2, B90.8, B90.9, J6, B52.0
Inflammatory (sarcoidosis, amyloidosis, pseudosarcomatous fibromatosis)	D86.0, D86.1, D86.2, D86.3, D86.8, D86.81, D86.82, D86.83, D86.84, D86.85, D86.86, D86.87, D86.89, D86.9, E85, E85.0, E85.1, E85.2, E85.3, E85.4, E85.8, E85.81, E85.82, E85.89, E85.9, M72.4
Other malignancies	C40, C40.0, C40.00, C40.01, C40.02, C40.1, C40.10, C40.11, C40.12, C40.2, C40.20, C40.21, C40.22, C40.3, C40.30, C40.31, C40.32, C40.8, C40.80, C40.81, C40.82, C40.9, C40.90, C40.91, C40.92, C48, C48.0, C48.1, C48.2, C48.8, C54, C54.0, C54.1, C54.2, C54.3, C54.8, C54.9, C55, C56, C56.1, C56.2, C56.9, C79.6, C79.60, C79.61, C79.62, C79.82

[a]Diagnoses include only specific diagnoses within an organ or other category that could be detected on breast MRI

© 2022 Ganguli I et al. *JAMA Network Open.*

eTable 8. Cascade Event Rates and Total and Out-of-Pocket Spending on Mammary Cascade Services among Screening Breast MRI vs Mammography Recipients in the 6 Months Following the Screening Test, Sensitivity Analysis including Mammograms as Cascade Events

	**Event rate per 100 members**		**Breast MRI cascade-attributable event rate per 100 members, 95% CI**
	**Breast MRI cohort N = 9,208**	**Mammogram cohort N = 9,208**	
**All mammary cascade events**	164.4	106.8	57.7 (51.9, 63.4)
**Mammary imaging tests**	48.5	24.8	23.7 (21.6, 25.9)
	**Spending per member**		**Breast MRI cascade-attributable spending per member, 95% CI**
	**Breast MRI cohort N = 9,208**	**Mammogram cohort N = 9,208**	
**Total spending on mammary cascade services**	$962	$337	$625 (591, 659)
**Out-of-pocket spending on mammary cascade services**	$102	$62	$40 (36, 45)

© 2022 Ganguli I et al. *JAMA Network Open.*

eTable 9. Total and Out-of-Pocket Spending on Mammary Cascade Services, Extra-Mammary Cascade Services, and All Services Among Screening Breast MRI vs Mammography Recipients in the 6 Months Following the Screening Test, Including Spending on the Index Event

		**Spending per member**		**Breast MRI cascade-attributable spending per member, 95% CI**
		**Breast MRI cohort N = 9,208**	**Mammogram cohort N = 9,208**	
**Mammary cascade services**	Total spending	$2,281	$575	$1,707 (1,674, 1,739)
	Out-of-pocket spending	$360	$55	$305 (297, 313)
**Extra-mammary cascade services**	Total spending	$2,116	$931	$1,185 (1,153, 1,216)
	Out-of-pocket spending	$397	$144	$254 (244, 263)
**All services**	Total spending	$7,227	$4,681	$2,546 (2,339, 2,753)
	Out-of-pocket spending	$837	$544	$293 (265, 320)

© 2022 Ganguli I et al. *JAMA Network Open.*
